# An application for identification and stratification psychological crisis among pandemic frontline healthcare workers

**DOI:** 10.1192/j.eurpsy.2022.365

**Published:** 2022-09-01

**Authors:** I. Szendi Md Habil, O. Bóna, T. Jenei, C. Kovács, Á. Nagy, K. Németh-Rácz, I.A. Török, E. Rudics, V. Dalos, V. Bilicki, M. Bácsfalvi, K. Téglás, Z. Szabó, E. Kelemen

**Affiliations:** 1 Kiskunhalas Semmelweis Hospital, University Teaching Hospital, Psychiatry Unit, Kiskunhalas, Hungary; 2 University of Szeged, Department Of Psychiatry, Szeged, Hungary; 3 University of Szeged, Department Of Software Development, Szeged, Hungary

**Keywords:** psychological crisis, Suicide, screening, Distress

## Abstract

**Introduction:**

The COVID-MENTA Screening Program was developed to monitor the mental health of frontline healthcare professionals and identify those at high risk for suicide at the Kiskunhalas Mobile Disease Control Hospital.

**Objectives:**

Our post hoc analysis aimed to investigate the association between psychological distress and suicide ideation based on passively collected data during the screening work.

**Methods:**

A sample of 50 healthcare professionals was analyzed from 167 participants in the COVID-MENTA Screening Program between the second and third waves of the COVID-19 pandemic. Data collection was performed during the breaks of healthcare professionals at work. Half of the group (*N=25*) perceived severe distress (scored > 5/10 on Distress Thermometer). The crisis monitoring application was based on Klonsky and May’s 3-step theory (2015) and was built by adapting the questions on the appropriate international scales (Psychache Scale, Beck’s Hopelessness Scale, Interpersonal Needs Questionnaire, Suicide Capacity Scale). The tool can stratify the current suicide risk into seven levels.

**Results:**

Spearman’s Rank Correlation was used for statistical analysis. There was a significant positive correlation between the psychological distress and the suicide risk (*r* (48) = 0,43, *p* < 0,01).

**Conclusions:**

Our findings supported the hypothesis of the study that the risk of suicide rises with the increase of the level of distress. The application has been proved effective in ecological conditions, helping in several cases to screen individuals currently at increased risk for suicide, allowing us to intervene in a timely and effective manner.

**Disclosure:**

No significant relationships.

